# Notes of M. Bernard’s Lectures on the Blood, with an Appendix

**Published:** 1855-05

**Authors:** 


Art. IV.—*Notes of M. Bernard's Lectures on the Blood, with an Appendix.* By Walter F. Atlee, M. D. Philadelphia: Lippincott, Grambo & Co. 1854.

This little volume has now been several months before the public, and much of the matter which it contains has been communicated to the profession from time to time by young American physicians resident in Paris. Some points, however, are entirely new, and the lectures of which it comprises the outline presents the most connected and complete view of the blood to be found in any recent work. We propose therefore to notice some of the leading topics of the work somewhat in detail, though conscious that in the months that have elapsed since its publication the subject has lost a portion of its novelty.

The blood is a product of the animal organism; it is fabricated by the living system, and is nearly the same in carnivorous animals and in those whose food is plants, but it is not identical in all parts of the same animal. The portal blood differs in its constitution from that of the hepatic vein. The effect of atmospheric air upon the color of this fluid is familiar to every one, but it is a mistake to suppose that the scarlet color imparted to it by oxygen is an indication of purity, since it has been shown by M. Bernard that the same color may be induced by gases excessively noxious. The oxide of carbon, one of the most deadly gases known, renders the blood red to a very high degree, which is the more remarkable since carbonic acid, which in composition is so nearly identical with this gas, is known to give it the venous color. What is more remarkable is, that this change is persistent, the blood once rendered red by the oxide retaining its vermillion hue under every exposure. Prussic acid also reddens the blood, and further change in color is prevented by its administration, the oxidizing property of the blood seeming to be at once arrested by the poison. A red color is, therefore, not always a sign of health. M. BERNARD once saw the blood flow so red from the vein of a typhoid patient that he supposed it, at first, to be arterial. The change from arterial to venous does not take place in the blood when nervous influence is suspended.

The temperature of the blood has generally been stated to be higher in the left than in the right ventricle, but M. BERNARD asserts that the reverse is the case, the difference amounting to about one degree Fahr., in favor of the right side of the heart. The maximum of temperature is in the portal vein. As the blood passes through the lungs it is cooled by the air. If the air "does so in the fingers why," asks M. BERNARD, "should it not do so in the lungs?"

The temperature of the blood cannot be much raised without fatal results. MAGENDIE showed by decisive experiments that animals in a heated atmosphere seemed to suffer very little, at first, but then died suddenly, and that their blood was hotter by from twelve to fifteen degrees Fahr. A higher temperature seems to be incompatible with life.

The quantity of blood varies somewhat in animals relatively to the weight of their bodies. In the dog it is one to four and a half; in the sheep one to five; in the cat one to five and a half; and in the rabbit one to six and a quarter. In a man weighing one hundred and forty-five pounds, M. BERNARD estimates the quantity of blood at thirty-two pounds. The quantity augments at each moment of digestion, as is proved by the rising of the mercury in a barometer attached to an artery; at the same time the liver and spleen swell, and are only made to diminish by secretion from the lungs and the kidneys. "In fasting an animal," says M. BERNARD, "consumes his blood, and dies just as in hemorrhage."

The blood flows with greater facility through the capillaries than a less viscid fluid would do. M. BERNARD injected water into the kidney of an animal by the renal artery, and found the tissue of the organ in a short time infiltrated and the circulation finally arrested. If in place of water serum of blood was injected no infiltration occurred. This fact affords an explanation of some interesting morbid phenomena. It is well known that the loss of blood will give rise to all the symptoms of plethora or capillary congestion, for which practitioners have in times past committed the serious mistake of resorting to blood letting. The headache, throbbing of the temples, intolerance of light, and other allied evidences of inflammation or congestion of the brain are the natural consequences of copious hemorrhages, and are relieved, not by depletion, but by remedies which increase the viscidity of the blood. "In order that the globules can circulate, the existence of fibrine is necessary. When you inject serum containing globules, or when the blood is defibrinized and the part is examined under a microscope, the globules are seen to be all at the bottom of the vessels, and at last the small vascular ramifications are blocked. The fibrine of the blood, then, keeps the globules from falling to the lowest part of the vessels; lighter than they are, it surrounds, envelopes, and separates them."

The rapidity of the circulation is affected by acids and alkalies, the latter increasing and the former retarding it, and other agents are also found to exercise an influence over the flow of the blood, as nitrate of potash and alcohol. The bearing of these facts upon pathology will be readily perceived.

Albumen, one of the most important constituents of the blood, modifies materially its solvent power, enabling it to dissolve many substances which naturally are not soluble in an alkaline liquid. It is the albumen which holds in solution the metallic matters in the blood, most of which, after a time, are deposited in some organ, as the albumen is appropriated. Thus antimony, lead, and arsenic find their way to the muscles, liver, and other organs.

The blood is the theatre of incessant changes. The phenomenon of calalysis takes place in it with the greatest facility, and it may be loaded with carbonic acid by injecting yeast and sugar into the vein of an animal, or rendered poisonous by the introduction at the same time of emulsine and amygdaline into the circulation.

It has lately been ascertained that the blood undergoes crystallization as well as coagulation when withdrawn from the body. This curious fact was discovered by OTTO FUNKE, and the blood employed by him being that of the spleen it was supposed to be peculiar to the blood of that viscus, but the experiments being multiplied it was seen that the vital fluid drawn from any part might be made to crystallize. It takes place more easily when a drop of water is added to the blood. When the crystals are made to form as large as possible, they are found on analysis to contain a large quantity of iron.

The fibrine of the blood, it would seem, may be increased by repeated blood-lettings, but it is not the same as the normal element; the fibres are shorter; it is less elastic, and resembles fibrine only in separating the globules from the rest of the blood. Macerated for twenty-four hours in warm water it is dissolved and presents the characters of an albuminous solution, giving support to the idea that fibrine is only transformed albumen.

The relation of the kidneys to the blood is one of great interest to the pathologist. These organs are prominent among the depurators of the economy. Bernard found that when substances generally elsminated by the kidneys were injected into the blood, they made their way into the stomach, and comparative anatomists have discovered that in the inferior animals, where there is no kidney, it is through this viscus that the eliminution takes place. The vomiting and derangement of digestion in persons affected with disease of the kidneys, it is probable, have their origin in the disturbing effect of urea in the alimentary canal.

The presence of sugar in the liver as a normal secretion, is a discovery for which physiological science is indebted to M. Bernard. Buchardat had found it in the blood of diabetic patients, but it was believed to be there as the result of disease. Magendie subsequently showed that it was always present in the blood, as a normal constituent, but he supposed that it came from sugar introduced from the exterior. Bernard has proved by a series of observations that it does not come from the aliment, but is generated by the liver whatever the character of the food used. It is not detected in the clot, and is destined to be eliminated, not as sugar, but as carbonic acid, the lungs destroying what the liver produces. The lower vena cava carries the sugar from the liver to the lungs through the right side of the heart, where it undergoes combustion. When but little sugar is made by the liver all is destroyed in traversing the lungs, but when more is formed there is an intermediate state, namely lactic acid, and when the quantity is more than one part to a hundred of blood, it may pass off by the kidneys and the patient become for the moment diabetic. At the commencement of diabetes, according to M. BERNARD, and also when recovery is taking place, sugar is only found during digestion.

It is from the iron contained in the blood, M. BERNARD holds, that the hematine possesses the property of absorbing oxygen, and it is only in the hematine that the iron is capable of fixing itself. Should there be no hematine, therefore, the simple administration of iron in anemic diseases would not be sufficient; the want of iron may depend upon the deficiency of hematine. Iron differs from the other metals occasionally detected in the blood in these circumstances, that it is only found in the globules, and never appears in the secretions, whilst, lead, copper, arsenic and the others localize themselves in the liver or muscles, or pass off with the secretions. No other metal but iron has been shown to be capable of playing a physiological part, though there is reason to believe that manganese may.

The effect of vascular plethora upon certain secretions has long been observed by practitioners. M. BERNARD shows by experiments upon dogs that the renal secretion is vastly increased by tying the arteries of all their limbs, by which the pressure upon the vascular tissue of the kidneys was raised from seventy-six to one hundred and twelve millimetres. The quantity of urine declines under venesection and is augmented by the injection of blood. In many cases, therefore, the excessive secretion of urine depends upon mechanical causes, but not in all; in some nervous affections there is an exaggerated amount of urine without any undue pressure upon the circulation. Pressure exerts no influence upon the salivary secretion, and the same is true of the function of many other glands.

"If these two secretions, the salivary and the urinary, be reflected upon, it will be seen that they could not act under similar influences, that they could not be regulated in the same way. The secretion of urine is constant, and must be under the influence of pressure, for it is connected with the purification of the blood. The salivary secretion is connected with certain acts of the digestion. The excretions must be distinguished from the secretions, which are not thrown out, whose active principles do not previously exist in the blood, but are formed in the organs, and are connected with certain acts of the economy. All such organs having these active principles in themselves, are not influenced by the external pressure, while those acting the part of a filter always are.

"The liver is probably between the two, between secretory and excretory. Suppose an obstacle, a calculus, for instance, to oppose itself to the excretion of the urine, the arterial pressure being resisted, the kidney will no longer secrete; the products usually excreted accumulate in the blood, and the accidents resulting from their non-elimination take place. When there is an obstacle to the elimination of the bile, those principles that certainly previously existed in the blood, are then retained, and the individual is jaundiced. But nothing similar can ever take place in the salivary glands; they become swollen, the products remain there, and never are found in the blood. Independently of these organs, there are many others that perform their functions differently as the pressure varies. As a general rule, the functions, cerebral, muscular, &c., diminish with the pressure. The action of coffee has thus been explained by some physiologists."

We have already seen that life is bounded by a limited range of temperature. A certain degree of heat is essential to all vital processes. Digestion, secretion, the metamorphosis of matters in the blood only go on when the vital heat is normal. The presence of oxygen favors these actions. Sugar disappears from the blood at the moment of the change to arterial, probably on account of the superabundance of oxygen. The changes that take place in the blood M. BERNARD does not hesitate to refer to *fermentation.* He says:—

"Arterial blood absorbs oxygen and abandons it, and this absorption varies and disappears, under the same circumstances as many fermentations, as, for instance, upon the lowering of the temperature. In making beer this is seen, and again we see that it ceases when the heat is raised above seventy-five degrees Centigrade, by the destruction of the organic principle producing the phenomenon. So in the blood, oxygen is absorbed in smaller quantities in the cold, and the effect is the same whether the blood be cooled artificially in a vase, or in the body. By raising the temperature the oxygen is found to be absorbed in proportion as the temperature rises. At forty-five degrees Centigrade, however, this principle in the blood, that absorbs oxygen is destroyed. Magendie has made many experiments on keeping animals in high or low temperatures; after long resisting the influence of heat, they finished at the end of a certain time by becoming warmer, but they never became hotter than forty-five degrees, when they died. It is precisely at this temperature that the blood ceases to absorb oxygen. This property is also lost when the temperature is very low. In the one case the blood is everywhere very red, both in the veins and the arteries; in the other, after the elevated temperature, on the contrary, it is everywhere black.

"There are then in the blood organic matters whose existence there is necessary for the absorption of oxygen. The phenomena which take place under their influence are the same as those of fermentation. All the liquids that act in digestion, act in this way; the gastric juice acts in virtue of an organic principle, and the pancreatic juice also. Pepsin has been found; it is purely and simply a ferment; experimenting with it, digestion will be found to commence at 10°, to increase to 40°, and then to diminish to 60°, when it ceases altogether. Without knowing this, Spallanzani made experiments on snakes, leaving them in the cold, and he found them to remain fifteen days without digesting. It is the same thing with the pancreatic juice, which ceases to act when the heat is so great as to coagulate it. This ferment acts in an alkaline medium, pepsin requires an acid one; that of the blood must be alkaline. Neutralize the gastric juice, and it no longer digests; it may be said that this is because it is the acid that digests, but take it alone and it does not. It is the *ferment* that digests, it must not be supposed to be the acid. The blood to absorb oxygen must be alkaline; it is normally so, but it is not the alkali that gives it this property, for a temperature of forty-five degrees does not destroy the alkalinity; it ceases then, because at that point the organic principle is destroyed. This special ferment has not as yet been isolated in the blood, but if none existed there, temperature would not exert the influence it does, upon its properties.

"Arterial blood never presents a buffy coat, as the venous is well known to do. This is easily observed in animals after death, when the white clot found in the right ventricle is never seen in the left. This might put us in the way of finding a reason for this buffy coat; it would appear as if, in traversing the lungs, a more intimate connection took place between the lymph and the blood."

The blood of the portal veins differs in many respects from that of other parts of the body,

"It is darker than other blood, the difference being very great when compared with arterial; though not so marked when compared with that from the hepatic veins, it was still very evident. It coagulates more slowly than any other blood, and the clot is diffluent, soft, scarcely solidified; it never presents a buffy coat, though it coagulates so slowly, a circumstance favorable to its formation. It absorbs a very great quantity of oxygen. In some experiments made the year before, M. Bernard observed that in this respect all bloods do not resemble each other. That of the vena portarum absorbed the most, from 20 to 25 per cent., that of the right ventricle, 18 to 20, of the jugular vein, 14 to 15, while the arterial blood only took up from 5 to 6 per cent. This property varies in different animals, the warm-blooded absorbing much more than the cold. Temperature also varies this absorbing power. This is enough, however, on this point for the present.

"The portal blood has to a very slight degree the property of becoming arterial in color. If venous blood be shaken in oxygen it becomes red. It is supposed that when oxygen is absorbed, that the blood is red, and when given up that it is black, and this is generally true, though not always so; the vein of the kidneys carries red blood. The clot of the blood from the vena portarum never becomes red on its surface; that of the hepatic veins does: the portal blood is the only one that never does become red on exposure to the air. This blood contains more iron than any other, and the iron may affect the amount of oxygen absorbed, but cannot affect the color. This fact seems to show that not only the iron must exist, in order that oxygen be absorbed, but also that it must be combined with something in order that the blood become red. There is more iron when the blood enters the liver than when it comes out; there is some elimination of iron in the bile. In the liver the blood loses iron, and there is also a diminution in its property of absorbing oxygen.

"As the portal blood possesses, then, properties very different from the arterial, it must have undergone a change in passing through the intestines. During digestion, other phenomena are seen; the blood in the vena portarum is much more abundant, and circulates much more freely. It contains more water, and many substances are found to have been acquired. Saccharine matters can be there under several forms, as cane-sugar, grape-sugar, and lactic acid. Fecula can never be absorbed as such; it must be previously changed; it must be rendered soluble, so as to be digestible; it changes to grape-sugar. Cane-sugar is partly changed to grape, and partly absorbed as it is.

"The portal is much less black during digestion than it was seen to be during abstinence, and its property of absorbing oxygen is diminished. Sugar has been shown by M. Bernard to diminish the property of the blood of absorbing oxygen.

"In regard to the albuminous bodies, the direct proof of their existing in greater quantity in the portal vein during digestion than during abstinence, is perfectly impossible. With sugar it is very easy, but with these it is different, as albumen and fibrine are always existing there. M. Bernard, however, made the following experiments that are very conclusive on this point. When he placed in the intestine, albumen, either of the egg or of serum, it disappeared, and none was found in the excrements. When, instead of that, he put some into a vein, he always found it in the urine; so that here it played the part of a foreign body, and was thrown off as such. What difference is there in the two conditions? In the one, being placed in the jugular vein, it went at once to the heart, but in the other it went previously through the liver, and it is here that the necessary modification takes place. That it is not from any action exerted by the intestines, is shown by this, that upon throwing it directly into the vena portarum, none made its appearance in the urine. None is ever found in the urine when it is forced to pass through the liver before entering the arterial circulation. The same thing is true of cane-sugar, which is always found again in the urine, after being thrown into the jugular vein, yet none is ever found there if it be thrown into the portal vein. Now, if the chyliferous vessels absorbed albumen, they would empty it into the subclavian vein, it would go into the arterial system before passing through the liver, and would be eliminated as a foreign body. It must hence be judged that albumen is not absorbed by the lymphatics, but that it enters together with the sugar into the portal vein.

"While the other *bloods* then remain about the same at all times, the blood in the vena portarum varies very much with what is eaten, and *above all* with the time. The blood in the arteries of herbivorous and of carnivorous animals is not essentially different; that in the vena portarum varies with the alimentation and with the period of digestion."

In regard to the source of sugar in the liver M. BERNARD says:—

"We can say then that the sugar is formed at the expense of the azotized or saccharine matters. The effect we have just seen fatty matters to have, and the special apparatus which has been demonstrated for their absorption, all go to show that they do not pass through the liver. The ciphers for the quantity of sugar formed when gelatin and fecula are given, are about the same as the ciphers representing the normal quantity in the blood.

"This sugar is chemically the same as glucose, but undergoes transformation with much more readiness. As the source of this sugar, fecula could only be called upon in animals having it in their food; carnivorous animals eating nothing capable of undergoing the transformation, nitrogenized matters must in them supply the material. In them, there is a diminution in the quantity of azotized matters in the blood, after traversing the liver, but a new principle, sugar, is found. As azote is not found in the sugar, it must have been separated in the liver, and it is found in the bile, by which means it is eliminated.

"This sugar is intimately connected with the functions of life. The quantity is normally the same in all animals, there being about 1-50 per cent. in the hepatic blood of both the carnivorous and the herbivorous. The greatest quantity M. Bernard has ever found was two per cent., and that was in a monkey who had eaten meat, butter, and carrots.

"This quantity of sugar must be mixed with all the blood of the body; already in the right ventricle, the proportion is much less, being only 0·69 per cent., as will be seen hereafter, in following the blood beyond the liver.

"In vertebrated animals, the sugar, being immediately emptied into the vena cava, is spread at once over the body; in the invertebrated, in place of going away by the hepatic veins, it goes back to the intestines by the biliary duct, and is absorbed there by a special apparatus before going to the respiratory organs.

"The quantity of sugar coming from the exterior, is very slight. When no saccharine matters are eaten, none is ever found in the portal blood, and when they are, you find only some traces of it; they may be taken up as lactic acid, &c. In the hepatic veins you have during digestion 1·50 per cent., and during abstinence one per cent., in *fresh* blood. The proportions given by Regnault are much more feeble; probably he was not aware of the great destructibility of this sugar. If it be not examined at once, a very great difference will be found; at the end of a few hours, in summer, when the destruction marches very rapidly, there is scarcely any. The sugar of diabetes is exactly the same as that of the liver, and it is also much more destructible than the others. being about twice as much so as ordinary glucose. The greater destructibility of the animal glucose, so to speak, over the vegetable, from which it cannot be distinguished by chemical tests, is shown by the following experiment. Under the skin of two rabbits, whose urine, previously tested, showed no trace of sugar, he injected, by means of the canula of a trocar, solutions of these two sugars. The experiment might be performed by injecting them into the veins, but in this case it might be objected to the result that too much had been suddenly introduced into the circulation, and on that account some would be found in the urine. When they are introduced under the skin, and thus made to undergo absorption before entering the circulation, this objection cannot be made. In the case in which the animal glucose was injected, no sugar was found in the urine when it was tested by the liquor of Barreswil, in the urine of the other rabbit it was. As said before, one is about twice as destructible as the other.

"The variations in the quantity of sugar have no relation whatever to those in the quantity of bile. Several modifications of the blood of the vena portarum occur in the liver, and they are independent of each other. It must not be thought that digestion, that is to say absorption, for absorption is the true digestion, commences immediately after deglutition. This absorption takes place several hours after eating. Five hours, then, after eating, when digestion is in full activity, we have the greatest quantity of sugar, and at this time, the flow of bile is feeble. It is when about ten hours have elapsed since eating that the flow of bile is greatest. This is perfectly conformable to what is seen in invertebrated animals, in whom, as has been said, the sugar returns by the biliary duct to the intestines. In them, at the time of digestion, you see a colorless liquor flowing by the duct; when digestion is finished you see a colored; the first is sugar, the second is bile."

We have read these Notes of the lectures of M. BERNARD with deep interest and are sure that the profession will thank Dr. ATLEE for the opportunity of becoming acquainted with the peculiar views of one of the first physiologists of the day.

